# Sleep Power Topography in Children with Attention Deficit Hyperactivity Disorder (ADHD)

**DOI:** 10.3390/children9020197

**Published:** 2022-02-03

**Authors:** Anna Castelnovo, Althea Lividini, Giulio Bernardi, Valdo Pezzoli, Giuseppe Foderaro, Gian Paolo Ramelli, Mauro Manconi, Silvia Miano

**Affiliations:** 1 Sleep Medicine Unit, Neurocenter of Southern Switzerland, Ospedale Civico, 6900 Lugano, Switzerland; mauro.manconi@eoc.ch; 2 Faculty of Biomedical Sciences, Università della Svizzera Italiana, 6900 Lugano, Switzerland; 3 University Hospital of Psychiatry and Psychotherapy, University of Bern, 3011 Bern, Switzerland; 4 Department of Medical and Surgical Sciences, University of Bologna, 40126 Bologna, Italy; althea.lividini@asst-santipaolocarlo.it; 5 MoMiLab Research Unit, IMT School for Advanced Studies Lucca, 55100 Lucca, Italy; giulio.bernardi@imtlucca.it; 6 Department of Pediatrics, Ospedale Civico, 6900 Lugano, Switzerland; valdo.pezzoli@eoc.ch (V.P.); Giuseppe.Foderaro@eoc.ch (G.F.); 7 Department of Pediatrics, San Giovanni Hospital, 6500 Bellinzona, Switzerland; GianPaolo.Ramelli@eoc.ch; 8 Department of Neurology, University Hospital, Inselspital, 3010 Bern, Switzerland

**Keywords:** EEG, spectral analysis, power, topography, sleepiness, maturation

## Abstract

Objective: Recent years saw an increasing interest towards sleep microstructure abnormalities in attention-deficit/hyperactivity disorder (ADHD). However, the existing literature on sleep electroencephalographic (EEG) power in ADHD is still controversial, often based on single electrode recordings, and mainly focused on slow wave activity (SWA) during NREM sleep. This study aimed to systematically investigate sleep power topography in all traditional frequency bands, in all sleep stages and across sleep cycles using high-density EEG (HD-EEG). Method: Thirty drug-naïve children with ADHD (10.5 ± 2.1 years, 21 male) and 23 typically developing (TD) control participants (mean age: 10.2 ± 1.6 years, 13 male) were included in the current analysis. Signal power topography was computed in classical frequency bands during sleep, contrasted between groups and sleep cycles, and correlated with measures of ADHD severity, cognitive functioning and estimated total sleep time. Results: Compared to TD subjects, patients with ADHD consistently displayed a widespread increase in low-frequency activity (between 3 and 10 Hz) during NREM sleep, but not during REM sleep and wake before sleep onset. Such a difference involved a wide centro-posterior cluster of channels in the upper SWA range, in Theta, and low-Alpha. Between-group difference was maximal in sleep stage N3 in the first sleep cycle, and positively correlated with average total sleep time. Conclusions: These results support the concept that children with ADHD, compared to TD peers, have a higher sleep pressure and altered sleep homeostasis, which possibly interfere with (and delay) cortical maturation.

## 1. Introduction

Attention-deficit/hyperactivity disorder (ADHD) is a common neurodevelopmental disorder broadly characterized by daytime symptoms of hyperactivity/impulsivity and inattention [1]. ADHD estimated prevalence in children and adolescence is around 5% worldwide [2,3,4,5]. ADHD high individual and societal impact have fueled intensive research over the last decades [2]. Nonetheless, the exact etiology of ADHD still remains largely unknown [6] and as a consequence, no objective/biological marker currently supports the diagnosis. Sleep problems are commonly reported by children-adolescents with ADHD and their parents in clinical settings [7]. This observation has led to a growing attention towards sleep [8] and its electroencephalographic (EEG) microstructure in ADHD [9,10]. Few quantitative EEG studies specifically investigated sleep power patterns in children with ADHD [9,11]. Older studies, which commonly focused only on one arbitrarily selected EEG channel, showed no abnormalities in the lower frequencies range (0.5–4.5 Hz) during non-rapid eye movement (NREM) sleep [12,13,14,15]. However, three later studies [16,17,18] have been performed in children with ADHD during sleep using high-density EEG (HD-EEG), which allows a higher level of spatial resolution of electrocortical activity in comparison to standard EEG. While two out of three studies found higher slow wave activity (SWA) over a centro-posterior cluster of electrodes [16,17], one found a global decrease in SWA in ADHD children compared to TD peers [18]. According to a recent meta-analysis, these apparently discrepant results might be explained by the negative association between SWA and both mean age and the use of medications [11]. 

Importantly, published results regarding frequency bands other than SWA were even more limited and contradictory, and were typically based only on the evaluation of a few scalp electrodes after cognitive demanding tasks. Previously mentioned HD-EEG studies focused only on SWA (mean signal power in the frequency range 0.5/1 to 4/4.5 Hz) during NREM sleep, since this parameter has a well-known role in synaptic, use-dependent plasticity and memory consolidation. However, all frequency bands have been found to undergo major modifications across typical development in both NREM and rapid eye movement (REM) sleep [9,19], and could thus reflect developmental alterations in children with ADHD. However, findings have been largely inconsistent across studies. These inconsistencies may be related to methodological issues and to the complexity and bidirectionality of the relationship between ADHD and sleep abnormalities [20,21]. Indeed, ADHD is a potential cause of sleep abnormalities per se [22,23], and sleep disorders are a potential source of ADHD-like symptoms [7,24]. In this regard, it is of utmost importance that quantitative EEG studies also consider PSG channels to assess major sleep comorbidities in ADHD.

In light of the above considerations, the aim of this study was to cover the aforementioned gaps in the literature, extending the analysis of common markers of ADHD using a previously collected dataset of overnight baseline HD-EEG/video-polysomnography (v-PSG) recordings [17]. In particular, while our previous study [17] focused on SWA in the first and last 60 min of NREM sleep, here we aimed to study all-night and all-frequency band power topography across sleep states (during both NREM stage 2—N2 and NREM stage 3—N3, REM sleep, and also pre-sleep-onset wakefulness), and across NREM sleep cycles, in order to explore candidate markers of disease.

## 2. Materials and Methods

This is an observational, prospective case-control single-center study carried out at the Neurocenter of Southern Switzerland on ADHD. All study procedures were reviewed and approved by the local Independent Ethics Committee “Comitato Etico Cantonale” (26 February 2015–n.2881), according to the regulatory requirements of Switzerland. All participants provided written consent before the study. 

### 2.1. Participants

Thirty children with a clinical diagnosis of ADHD and 23 healthy control peers were included for analysis. This dataset overlaps with the one described in a recent publication by our group [17]. Demographics and clinical information are summarized in Table 1.

#### 2.1.1. Patient Group

Drug-naïve children with ADHD were recruited consecutively at the local Pediatric Department (in Lugano and Bellinzona) from April 2015 to May 2016. Each patient was evaluated by both a pediatrician and by a pediatric neuropsychiatrist (SM). The diagnostic protocol included a detailed medical history with both children and their parents, a neurological examination, a semi-structured psychiatric interview, i.e., the Schedule for Affective Disorders and Schizophrenia for School-Age Children Present and Lifetime Version (K-SADS-PL) [25], a paper-and-pencil version of the Conners’ Parent Rating Scale—Revised (CPRS-R) [26] filled in by parents, and the Wechsler Intelligence Scale for Children—IV (WISC-IV) [27] and the Neuropsychological Developmental Assessment—Second Edition (NEPSY-II, a standardized neuropsychological battery for children) [28], administered to children with ADHD by a neuropsychologist and cognitive psychotherapist.

Inclusion criteria were: (1) a formal diagnosis of ADHD according to Diagnostic and Statistical Manual of Mental Disorders—5th Edition (DSM-V) criteria [29]; (2) age between 8 and 14 years. Exclusion criteria were: (1) a comorbid diagnosis of autistic spectrum disorder (ASD); (2) an intelligence quotient <70; (3) other known major neurological conditions; (4) previous treatment with stimulants or other medications used to treat ADHD.

The final group included 30 Caucasic research participants (mean age: 10.5 ± 2.1 years, range: 8–13 years, 21 male); of them, 22 were diagnosed with a combined ADHD presentation, 6 with a predominantly inattentive and 2 with a predominantly hyperactive presentation [17]. 

All research participants underwent a complete sleep assessment, which included a 1-week actigraphy recording, a nocturnal video-polysomnography (v-PSG) with extended EEG monitoring, and a multiple sleep latency test (MSLT) the day after the v-PSG. Good quality, all-night recordings were available for 29 participants. For one subject only the first cycle (NREM sleep plus a few epochs of REM sleep) was available and used for the current analysis. 

According to a previous classification of the same group of patients, children with ADHD could be divided into 5 different phenotypes: (1) epileptic EEG abnormalities (*n* = 10); (2) sleep onset insomnia (*n* = 5), based on a reported sleep latency > 20 min, for more than 3 times per week and more than three months; (3) periodic limb movements (PLM) >5 events/hour with no associated restless leg syndrome (RLS) (*n* = 8); (4) obstructive sleep apnea-hypopnea syndrome (OSAS), based on an apnea-hypopnea index (AHI) >1 events/hour and the presence of at least one of the following clinical features: snoring, labored sleep breathing, excessive daytime sleepiness (*n* = 15); (5) narcoleptic-like phenotype (*n* = 4), characterized by excessive daytime sleepiness as defined by an MSLT <8 min, and/or 2 sleep-onset REM-sleep periods at MSLT. For more details on the clinical features of this group, see Miano et al. [17].

#### 2.1.2. Control Group

Twenty-five TD children were recruited by e-mail and word-of-mouth among all employees of the Civic Hospital of Lugano. A physician board-certified in both Pediatric Sleep Medicine and Child Psychiatrist (SM) thoroughly interviewed children and their parents to screen for any known sleep disorder, neuro-psychiatric comorbidity, or any medical condition affecting sleep. Selected children were then referred to the sleep laboratory for a sleep v-PSG with extended EEG monitoring. 

Data collected from 2 participants were lost due to storage failure. Therefore, recordings from 23 participants were eventually used in the analysis (mean age: 10.2 ± 1.6 years, range: 8–13 years, 13 male). The control group did not significantly differ from the patient group for age (t_(51)_ = −0.66, *p* > 0.05, independent-samples 2-tailed *t*-test) and sex (χ^2^ _(1, *n* = 53)_ = 1.1, *p* > 0.05, Chi-square test for independence with Yates Continuity Correction). Healthy control participants were also screened for sleep breathing disorders and PLM during sleep. None of them had symptomatic OSAS or an AHI >5 events/hour or reached the criteria for a PLM disorder or RLS.

### 2.2. Sleep Recordings

All participants underwent an in-laboratory overnight HD-EEG recording (256 channels; Electrical Geodesics Inc., Eugene, OR, vertex-reference, 250 Hz), coupled with traditional v-PSG [30]. Lights out was consistent with the participants’ average bedtime, and wake-up time was between 6 and 7 am for all participants.

Sleep stages and sleep events were scored according to standard criteria by two board-certified sleep physicians using the Embla^®^ Remlogic Software (Neurolite), based on 30-s epochs for 6 bipolar re-referenced EEG channels (F3/A2, F4/A1, C3/A2, C4/A1, O1/A2, O2/A1), electrooculogram (EOG), and submental electromyography (EMG) [30]. 

### 2.3. EEG Signal Power in NREM Sleep, in REM Sleep and Wake before Sleep Onset

Before spectral analysis, data were pre-processed according to standard routines for HD-EEG. All EEG signals and other relevant information (including sleep scoring) were imported and analyzed in MATLAB (The MathWorks Inc., Natick, MA, USA). Each signal was first-order high-pass filtered at 0.1 Hz (IIR filter reproducing a single resistor capacity) and subsequently band-pass filtered (0.5–45 Hz, Kaiser window-based FIR with zero-phase distortion). Data epochs corresponding to NREM sleep N3 and N2, REM sleep and wake before sleep-onset (see below) were extracted and pre-processed separately. An interactive open-source tool for data visualization and data-cleaning (https://github.com/CSC-UW/csc-eeg-tools.git, accessed on 2 February 2021) was used to visually inspect data in MATLAB. Channels with clear artifacts were removed (and later recovered through interpolation; see below), while data segments containing artifacts affecting the majority of channels were marked as “bad” and not considered in subsequent analyses. Channels displaying a sharp difference in power relative to neighboring channels upon inspection of power spectra and topographic power maps were additionally removed. Quiet windows of wakefulness available in the time period between the end of electrode net setup and the beginning of the overnight recording (from 10 to 50 min before actual sleep onset) were selected. After the removal of artifactual data segments, a total of 8–16 min (M = 12.3, SD = 3.6) of wakefulness was retained for 27 patients and 18 control subjects. Three patients and 4 control subjects did not have artifact-free segments of wakefulness before sleep onset longer than 5 min and were therefore excluded. Independent Component Analysis (ICA) was performed on both sleep (REM and NREM) and wake data, in order to remove ocular, electrocardiograph, sweating, epileptic spikes and remaining muscular artifacts using EEGLAB routines [31]. Only ICA components with characteristic activity patterns typical of these artifactual activities were removed. Subsequently, the removed, bad channels were interpolated using spherical interpolation.

Spectral analysis was performed using all artifact-free 6-s epochs (Welch’s averaged modified periodogram with a Hamming window, 8 segments with 50% of overlap) on the average-referenced signal. For topographic analysis, average signal power (across epochs) was computed for 6 classical frequency ranges [32]: delta/SWA (1–4 Hz), Theta (4–8 Hz), Alpha (8–12 Hz), Sigma (12–16 Hz) Beta (16–25 Hz), low Gamma (25–40 Hz). Both topographic maps of absolute average-referenced and normalized data (z-score across channels of the same participant) were examined. NREM sleep (more specifically stages N2 and N3, taken together and separately) and REM sleep all-night power maps as well as wake before sleep onset power maps were compared across groups (ADHD versus TD children) for all frequency bands. 

### 2.4. NREM Sleep Homeostatic Regulation

Additional between-groups analyses were performed to compare the first, second and third sleep cycles separately, for the range of frequencies that significantly differed between groups in the all-night analysis. Of note, we specifically focused on the first three sleep cycles because this was the maximum number of cycles represented in the majority of our participants. In order to evaluate whether the physiological decline of low-frequency bands was preserved or altered in ADHD versus TD children, we also compared power in these frequencies across cycles and between groups. 

### 2.5. Correlations between Power and Clinical Variables

The association between EEG power and clinical variables in the ADHD group was investigated for the frequency range that showed a significant difference between ADHD and TD children in the region of interest (ROI) of interest. Selected clinical variables were age, habitual total sleep time (TST) as estimated by parents and average sleep latency at the MSLT (as measures of sleep pressure), the global ADHD scores at the Conners Rating Scale, and global intellectual ability measured with the WISC-IV.

### 2.6. Statistical Analysis

Statistical between-group comparisons of demographic and polysomnographic variables, as well as power spectra, were performed using unpaired 2-tailed *t*-tests, Mann–Whitney U tests, or χ^2^ tests, as appropriate. Normality of data and homogeneity of variance were evaluated using the Shapiro/Wilk’s test and Levene’s test, respectively. Comparisons of scalp power maps were performed separately for each frequency band. At the scalp level, we corrected for multiple comparisons using a non-parametric cluster-based permutation test [33], as described in previous work [34,35,36,37,38,39]. Specifically, for each performed test, a null distribution was generated by randomly shuffling the group-label of each subject for comparisons. At each iteration of the permutation procedure, the test-statistics was computed for each electrode and the size of the largest significant electrode-cluster (uncorrected *p* < 0.05) was stored in a frequency table. Given the impracticality of computing all possible data re-combinations, the full null distribution was approximated using 10,000 iterations. Finally, the 95th percentile (5% significance level) was used as the critical cluster-size distribution threshold. Given the possible impact of age on these variables, an additional analysis was conducted with age introduced as a covariate. For the sake of simplicity, only results without using the covariate are detailed, except for cases where a discrepancy between the two analyses was observed. We did not correct for the issue of multiple testing across different comparisons (across bands and stages) due to the exploratory nature of this study.

In order to explore stage differences in average power comparisons, a mixed model analysis of variance (ANOVA) was used to determine the interaction effect between groups (ADHD and TD children) and sleep stages (N2 versus N3). In order to explore differences in the homeostatic regulation of sleep microstructural features, a mixed model ANOVA was used to assess the interaction effect between group (ADHD and TD children) and sleep cycle (first, second and third cycle), for the average power ROI identified by the cluster test analysis. 

Correlation between power values and clinical variables in the patient group were performed using Spearman’s correlation. As we performed 5 different correlations, the level of significance was adjusted for multiple comparisons (Bonferroni’s correction, 0.05/5 = 0.01). 

Statistical analyses were performed in MATLAB.

## 3. Results

### 3.1. EEG Signal Power in NREM Sleep, REM Sleep and Wake before Sleep Onset

#### 3.1.1. NREM Sleep

During whole night NREM sleep children in the ADHD group showed a widespread, significant increase in absolute Theta power (cluster size = 124, *p* < 0.05) relative to the healthy control group (see Supplementary Figure S1). The increase was observed in most electrodes, with the notable exception of frontopolar ones.

No significant differences between children with ADHD and control participants were found in other frequency ranges and for normalized power except for a small frontal Beta cluster (cluster size = 18, *p* < 0.05), which was not confirmed after the introduction of age as covariate.

As there was a significant difference between the two groups (ADHD and TD) in the AHI, we also performed an exploratory analysis within the ADHD group between patients with (*n* = 15) and without (*n* = 15) the obstructive hypopneas/apneas phenotype. We could not observe any difference between these two groups in any frequency band (see Supplementary Figure S2).

**Figure 1:**
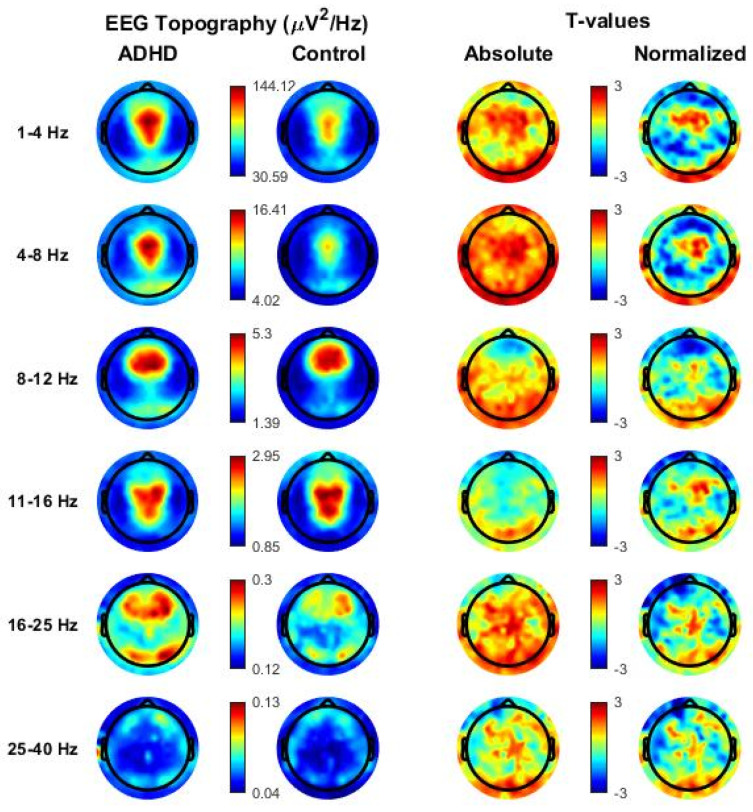
Topographical distribution of all frequency bands based on whole-night non-rapid eye movement (NREM) sleep stage 2 (N2) in the attention-deficit/hyperactivity disorder (ADHD) group and in the healthy control group. Values are color-coded and plotted on the planar projection of the hemispheric scalp model. Rows: frequency bands of interest. First and second column: average N2 sleep EEG topographies across frequency bands for children with ADHD and healthy control matches, respectively. Maxima are shown in red, minima in blue. Third and fourth column: single electrode *t*-value (2-tailed, unpaired) maps for the comparison between patients with ADHD and control subjects in terms of absolute and normalized (using the z-score across all electrodes) power, respectively. Blue color: decrease in EEG power in patients with ADHD relative to healthy controls (ADHDs < controls), red color: increase in EEG power in patients with ADHD relative to healthy controls (ADHD > controls). White circles indicate significant electrodes (*p* < 0.05 cluster-size correction).

In N2 sleep, many individual electrodes showed significantly higher absolute power values in the ADHD group relative to the control group, especially in low-frequency ranges (SWA, Theta, Alpha; *p* < 0.05, uncorrected). However, no significant effects were found for both absolute and normalized power maps (see Figure 1) after multiple comparison correction.

**Figure 2:**
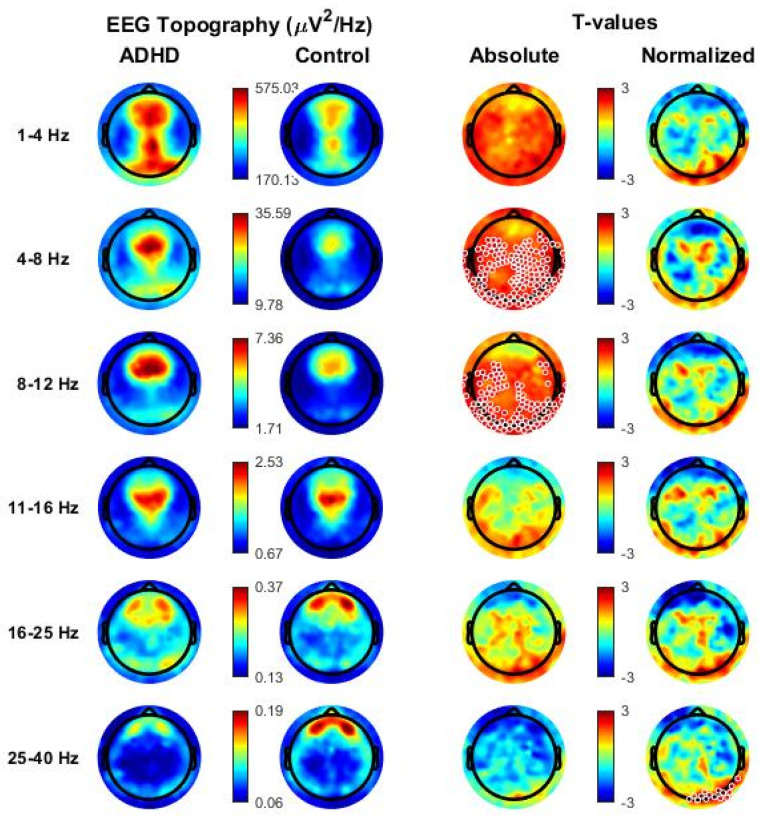
Topographical distribution of all frequency bands based on whole-night non-rapid eye movement (NREM) sleep stage 3 (N3) in the attention-deficit/hyperactivity disorder (ADHD) group and in the healthy control group. Values are color-coded and plotted on the planar projection of the hemispheric scalp model. Rows: frequency bands of interest. First and second column: average N2 sleep EEG topographies across frequency bands for children with ADHD and healthy control matches, respectively. Maxima are shown in red, minima in blue. Third and fourth column: single electrode *t*-value (2-tailed, unpaired) maps for the comparison between patients with ADHD and control subjects in terms of absolute and normalized (using the z-score across all electrodes) power, respectively. Blue color: decrease in EEG power in patients with ADHD relative to healthy controls (ADHDs < controls), red color: increase in EEG power in patients with ADHD relative to healthy controls (ADHD > controls). White circles indicate significant electrodes (*p* < 0.05 cluster-size correction).

In N3 sleep, a widespread significant increase in absolute Theta (cluster size = 143, *p* < 0.05) and Alpha (cluster size = 99, *p* < 0.05) was observed in the ADHD group relative to the healthy control group (see Figure 2). Differences were again observed in most central, parietal, temporal and occipital electrodes, and only spared frontopolar electrodes. When age was introduced as covariate, also the SWA range remained significant after multiple comparison correction (SWA range: cluster size = 134, *p* < 0.05, Theta range: cluster size = 197, *p* < 0.05, Alpha range, cluster size = 46 *p* < 0.05). 

No significant differences between ADHD and control children were found in other frequency ranges and for normalized power except for a small posterior Gamma cluster (cluster size = 15, *p* < 0.05), which was not confirmed after the introduction of age as covariate. It is however noteworthy that several individual posterior electrodes showed relatively higher levels of normalized SWA in ADHD, compared with control children (*p* < 0.05, uncorrected), in line with previous research.

**Figure 3:**
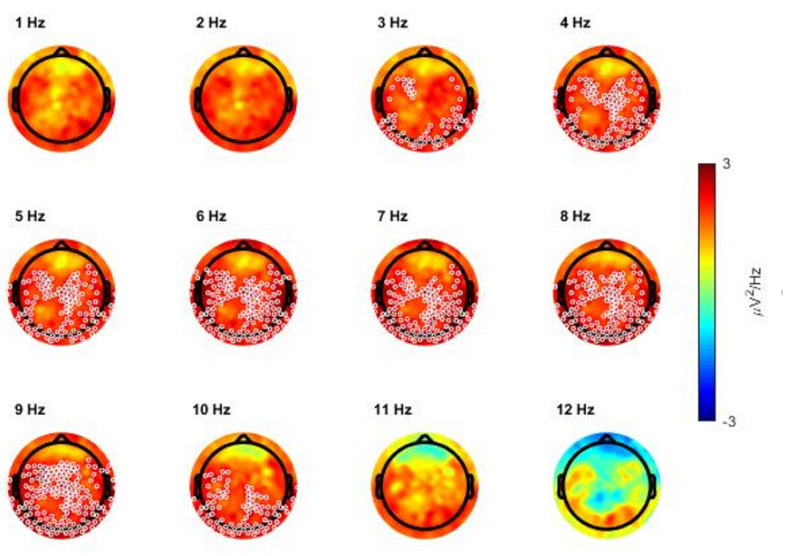
Topographical distribution of the comparison (single electrode *t*-value, 2-tailed, unpaired) between absolute power values of the attention-deficit/hyperactivity disorder (ADHD) group and of the healthy control group during whole-night non-rapid eye movement NREM sleep stage 3 (N3), represented per bin of frequency.

Additional analyses were performed to better characterize the low-frequency increase observed during N3 sleep in ADHD relative to control children. In particular, we first compared the EEG power spectra (power spectral density averaged across all scalp channels) of the two groups and found a significant difference between 2.7 and 9.8 Hz (Supplementary Figure S3). Consistent results also emerged from the analysis of topographical power maps when power was computed in 1 Hz bins (Figure 3). In particular, we found significant clusters with higher power in ADHD relative to control children in frequency bins from 3 to 10 Hz.

Each map represents the comparison for one bin of frequency centered on the value indicated above each map. Values are color-coded and plotted on the planar projection of the hemispheric scalp model. A lower EEG power in patients with ADHD relative to healthy controls (ADHDs < controls) is represented in blue, a higher power (ADHD > controls) in red. White circles indicate significant electrodes (*p* < 0.05 cluster-size correction). 

Interestingly, observed differences involved most low-frequencies below 10 Hz, with the notable exception of frequencies below 2.5 Hz (Supplementary Figure S4). When slow (0.5–2.5 Hz) and fast SWA (2.5–4 Hz) were considered separately, a cluster of significance could be observed only in the fast SWA range (cluster size = 31, *p* < 0.05). 

**Figure 4:**
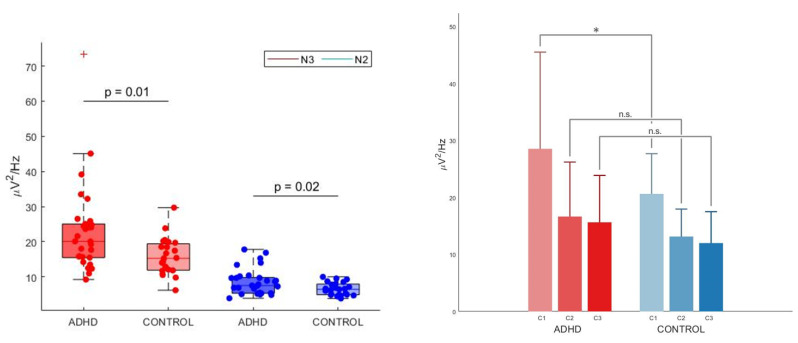
Left panel—Absolute spectral density averaged across channels within the significant Theta cluster in the attention-deficit/hyperactivity disorder (ADHD) and the control group in non-rapid eye movement (NREM) sleep stage 3 (N3) and NREM sleep stage 2 (N2). Red dots represent N3, blue dots N2. The bottom and top of each boxplot are the 25th and 75th percentiles of the sample, respectively. The distance between the bottom and top of each box is the interquartile range. The green line in the middle of each box is the sample median. The whiskers extending above and below each box go from the end of the interquartile range to the furthest observation within the whisker length. Observations beyond the whisker length (more than 3 times the interquartile range away from the bottom or top of the box) are marked as outliers. The cross (+) represents an outlier. The mean difference was significant between the ADHD group (N3: M = 22.90, SD = 12.65, N2: M = 8.60, SD = 3.73) and the control group (N3: M = 15.77, SD = 5.22, N2: M = 6.54, SD = 1.81) for both N3 (*p* < 0.05, also after the removal of the outlier in the ADHD group, Cohen’s d = 0.70, meaning a medium effect size, independent samples *t*-test) and in N2 (*p* = 0.018, Cohen’s d = 0.68, meaning a medium effect size, independent samples *t*-test). There was a significant interaction effect (*p* < 0.05) between stages (N3 versus N2) and groups (ADHD versus control) at a mixed between-within ANOVA model (not shown in the figure). Right panel—Absolute spectral density averaged across channels within the significant Theta cluster in the attention-deficit/hyperactivity disorder (ADHD) and the control group in different sleep cycles (first, second and third cycle). X-axes: time expressed in cycles, Y-axes: average power values in selected frequency range of 3–10 Hz, expressed in µV/Hz^2^. Red bars: ADHD group. Blue bars: control group. C1: first cycle, C2: second cycle; C3: third cycle. There was a significant difference in 3–10 Hz power of the significant cluster of channels for the ADHD group (M = 28.55, SD = 16.97) and the control group (M = 20.63, SD = 7.06) in N3 of the first sleep cycle: t(51) = 2.10, ADHD > controls, *p* < 0.05, Cohen’s d = 0.61, meaning a medium effect size, independent-samples *t*-test, two tailed). n.s.: not significant. (*): significant comparison.

We then defined as ROI the cluster of electrodes that survived multiple comparison correction in the frequency range between 3 and 10 Hz. In order to investigate whether the low-frequency difference observed in N3 sleep (3–10 Hz range) was specific for this stage or also extended (and to which degree) to N2 sleep, we used an ANOVA model with group (ADHD versus TD) and stage (N2 versus N3) as factors to analyze the average signal power in the region of interest. There was a statistically significant two-way interaction between group and stage: F_(2, 50)_ = 6.139, *p* < 0.05, Huynh–Feldt correction for Epsilon > 0.75, partial eta squared = 0.109, see Figure 4, left panel). As expected, there was a significant effect of stage on power for both ADHD and TD groups (N3 > N2, *p* < 0.001, *p* Bonferroni-adjusted < 0.001 for both groups). Group comparisons were significant for both N2 (ADHD > TD, *p* Bonferroni-adjusted < 0.05) and N3 (ADHD > TD, *p* Bonferroni-adjusted < 0.05).

#### 3.1.2. REM Sleep Power

No differences were found between groups (ADHD versus TD) in terms of absolute or normalized power maps in REM sleep (see Supplementary Figure S5). No clusters of significance were found after multiple comparison correction also when considering the ROI emerged in NREM sleep analysis (data not shown). 

#### 3.1.3. Wake before Sleep Onset Power

No differences were found between groups (ADHD versus TD) in terms of absolute or normalized power in wake before sleep onset except for a small posterior Gamma cluster in normalized power maps (cluster size = 14, *p* < 0.05), which disappeared after the introduction of age as a covariate (see Supplementary Figure S6). No significant clusters were found after multiple comparison correction in 3–10 Hz power the ROI emerged from NREM sleep analysis (data not shown).

### 3.2. NREM Sleep Homeostatic Regulation

We first conducted separate group-level comparisons using 3–10 Hz power in the ROI of interest during the first, second and third cycle of N3 sleep, separately. Four research participants were removed from this analysis because they did not have N3 in the third cycle. 

A 2-way mixed ANOVA used to investigate potential inter-group differences in 3–10 Hz power across cycles in the previously defined ROI revealed an interaction effect between group (ADHD versus TD) and cycle (cycle 1, cycle 2, cycle 3), within our significant cluster of channels: F_(1, 46)_ = 3.318, *p* < 0.05 (Huynh-Feldt correction for Epsilon > 0.75, partial eta squared = 0.066). Pairwise comparisons showed that power was significantly different between groups in cycle 1 (ADHD >TD, *p* <0.05), but not in cycle 2 and 3 (although this effect did not stand multiple comparison correction with Bonferroni’s adjustment, see Figure 4, right panel). There was a statistically significant effect of cycle on power for each group (*p* < 0.05 for both groups) which confirmed a physiological homeostatic decline in N3 power in both ADHD and TD. The pairwise comparisons cycle 1 versus cycle 2 and cycle 1 versus cycle 3 were significantly different for both groups (*p* < 0.05). 

### 3.3. Correlation Analysis between Low-Frequency Activity and Clinical Variables

**Figure 5:**
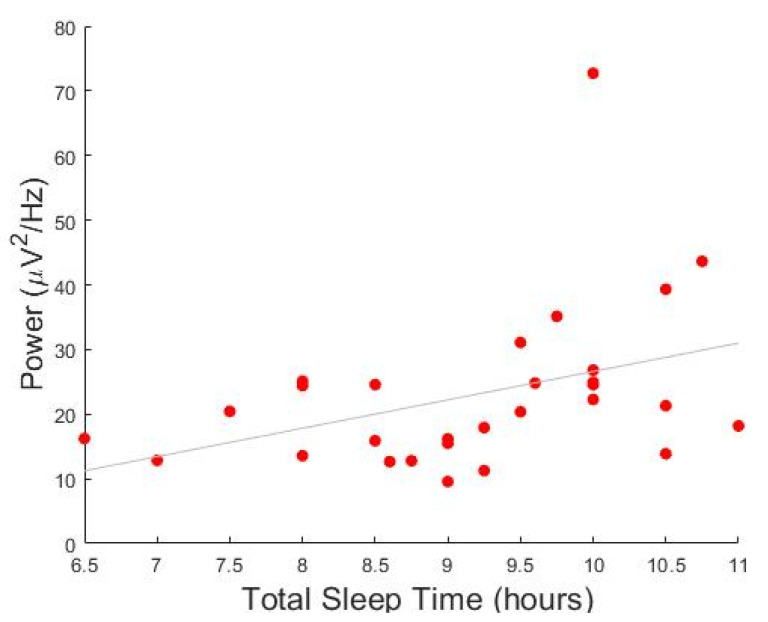
Correlation between power and total sleep time in children with attention deficit/hyperactivity disorder (ADHD). *Y*-axis represents the average power of all significant channels in the 3–10 Hz cluster. *X*-axis represents the habitual total sleep time estimated by parents.

Given our observation of higher 3–10 Hz activity in ADHD relative to TD children during N3 sleep, we investigated whether this low-frequency power in the ROI of interest was correlated with demographic and clinical variables of children with ADHD. We found a significant negative correlation between low-frequency power and age, so that older children were characterized by lower signal power (*p* < 0.001, r = −0.515; Figure 5). In addition, we observed a significant positive correlation with the total sleep time as estimated by the parents (*p* < 0.001, r = 0.490). This correlation remained significant when the effect of age was partialled-out. No significant correlations were found between signal power in the 3–10 Hz range and MSLT mean sleep latency, overall ADHD symptoms measured by the Conners’ scale (*p* > 0.001, r = 0.14), and the WISC-IV (*p* > 0.001, r = 0.29). 

## 4. Discussion

### 4.1. General Overview

This study expands our previous findings on normalized sleep power topography in ADHD children, which pointed to a relative increase of SWA power over centro–parietal–occipital regions in these subjects compared to TD children [18]. Here, we investigated systematically both absolute and normalized power density maps in all traditional frequency bands and in all sleep stages in the same dataset of drug-naive children with ADHD and healthy control peers, collected with HD-EEG coupled with a complete PSG monitoring [17]. Of note, contrary to previous studies investigating quantitative EEG differences in ADHD and TD children, this dataset was specifically tailored to investigate sleep problems in ADHD children but additionally demonstrated a higher prevalence of past and/or current sleep disorders in the ADHD children group compared to the control group. Despite the heterogeneity of sleep disorders in this patient population [17], our results consistently revealed a widespread increase in low-frequency activity, between 3 and 10 Hz, during NREM sleep in ADHD compared to TD children, but not during REM sleep and wake before sleep onset. We interpreted this effect as the common end-stage result of different sleep disorders on brain development. Such a difference involved the upper SWA range and peaked in the Theta range, encompassing a wide centro-posterior cluster of channels, sparing only fronto-polar regions. In addition, the difference was maximal in stage N3, although a similar trend could also be observed in N2 sleep. Between-group differences were more marked in the first sleep cycle, suggesting an increased homeostatic pressure in ADHD children. Average power values in the 3–10 Hz frequency range were positively correlated with estimated average total sleep time. 

Overall, current results widen the perspective from previous sleep HD-EEG studies which focused exclusively on SWA in NREM sleep and clearly showed that all lower frequency bands (and mainly Theta band) are altered in children with ADHD during NREM sleep, paralleling findings during daytime “rested” wakefulness [40].

### 4.2. Interpretation of Results

This broad-band effect likely reflects the fact that the cut-offs of frequency bands, although based on the visual inspection of EEG activity, frequently do not correspond to the functional meaning of EEG rhythms [41]. Indeed, all lower EEG frequency bands are all homeostatically regulated in humans [42], meaning that their power increases with sleep deprivation and gradually dissipates during a good night of sleep [43,44]. Furthermore, the power of EEG slow frequencies during sleep varies significantly with brain maturation [40]. More specifically, global SWA activity during NREM sleep significantly increases in the first decade of life and then decreases during the second decade of life, both in cross-sectional and longitudinal studies [19,45,46,47]. Theta activity undergoes a similar trajectory, although its peak and decline begin significantly earlier compared to SWA (starting approximately at 9 years of age) [45]. The Alpha band has been less investigated, but the limited existing literature overall suggests a reduction in the Alpha band in the second decade of life [19,47,48]. Taken together these data contrast with the maturational trajectory of higher frequency bands [49] which seems to be less affected (Beta/Gamma) by age or to be modulated differently, as in the case of Sigma activity, following a bimodal curve with a first peak in the slow spindle range after the age of two years and a second, centro-parietal peak at high-Sigma frequencies during adolescence [42,50,51].

Therefore, an abnormality of lower EEG frequency bands in ADHD might both reflect a delay in brain maturation and/or an alteration in sleep homeostasis.

Delay in brain maturation—Higher power values from 3 to 10 Hz sparing more frontal areas may be consistent with ADHD children presenting a “younger” (i.e., less “mature”) power pattern compared to their peers. This hypothesis is reinforced by the typical clinical evolution of the disorder, as symptoms tend to improve with age [52]. However, it should be noted that the results from previous longitudinal and cross-sectional, low-density EEG studies [47,53], reported a significant decrease with age of EEG power in the delta/Theta/Alpha bands during both REM sleep and NREM sleep [47]. In this respect, consistency with the maturation delay hypothesis would imply a dissociation between NREM and REM sleep, with only maturational aspects reflected by NREM low-frequency activity actually altered in ADHD relative to TD children. 

Sleep homeostasis impairment*—*At the same time, high levels of low-frequency activity may reflect a higher sleep pressure and an abnormal homeostatic process in children with ADHD. The homeostatic process is fundamental for brain plasticity [54] and is expressed by changes in the EEG power spectrum between the first and the second half of the night, mainly in the 1–9 Hz range [55], in face of a stable power topography. Our ADHD group displayed a specific increase in this frequency range, in the first sleep cycle, which rapidly and progressively dissipated throughout subsequent cycles. Interestingly, our results on SWA, the most commonly studied index of sleep pressure, are consistent with recent findings in animal models showing that higher-frequency SWA (2.5–3.5 Hz) respond to sleep loss with high initial power and fast, discontinuous decay during recovery sleep, while lower-frequency SWA (0.5–2.75 Hz) seems unrelated to time-spent-awake [56]. The increased sleep pressure in children with ADHD might be linked to the fact that subjective sleep complaints are common in persons with ADHD. Sleep disturbances were indeed confirmed objectively in our ADHD sample in a previously published paper [17]. It can be further speculated, on the basis of a positive correlation between estimated total sleep time and increased power in the 3–10 Hz range, that intrinsic abnormalities and/or underlying sleep disorders prevent these children from obtaining a fully restorative sleep, leading to a compensatory (but insufficient) increase in total sleep time. Finally, sleep abnormalities may impair neurodevelopmental and cortical maturational processes that are associated with sleep [57,58].

### 4.3. Limitations and Future Perspectives

To our knowledge, the analyzed dataset is the first that combined both quantitative EEG analysis and a full sleep evaluation (clinical interview, actigraphy, PSG and MSLT). This allowed us to discover an otherwise overlooked high prevalence of sleep disturbances in children with ADHD (as sleep assessments are not required for a formal diagnosis of ADHD). Notably, we found consistent results despite the heterogeneity of sleep disorders in our population. However, this heterogeneity prevented the possibility to explore the specific contribution of each sleep disorder to quantitative EEG findings. Although our sample was larger than those considered in many previous EEG studies in children with ADHD, increasing the sample size in future studies is warranted to investigate the impact of specific individual variables, such as gender, pharmacological treatment or sleep disturbances. Another potential limitation of this study was the lack of a detailed neuropsychological assessment and objective evaluation of daytime sleepiness/total sleep time (using MSLT and actigraphy) in the control group. This possible confounding bias should be ruled out in future studies. However, it should be noted that all control subjects underwent a sleep and neuropsychiatric screening interview and no sleep, cognitive or psychiatric complaint emerged from this clinical assessment.

Protocols involving a parallel acquisition of neurophysiological (EEG), anatomical (magnetic resonance imaging) and cognitive (executive functions) variables would ensure a deeper understanding of the neurobiological underpinnings and meaning of the findings we presented in this study. Furthermore, a longitudinal interventional study design (targeting sleep problems and/or ADHD per se with methylphenidate), would also help to elucidate both the causal relationship of sleep abnormalities on ADHD symptoms, as well as the impact of treatment strategies on ADHD prognosis. 

## 5. Conclusions

In summary, we described for the first time, sleep power topography in ADHD on a broad range of frequencies during both NREM and REM sleep and across sleep cycles.

We found a global increase in low frequencies (high SWA, Theta and low Alpha, from 3 to 10 Hz) in NREM sleep of drug-naïve children with ADHD compared to TD children. This effect was specific for NREM sleep which could not be observed during REM sleep and pre-sleep-onset wakefulness and was more prominent in N3 in the first sleep cycle.

These findings reinforce the link between sleep neurophysiological abnormalities and ADHD. Moreover, our study found support for two recognized hypotheses regarding ADHD pathogenesis and suggests that they might not be in open contradiction: on one hand, cortical maturation seems to be delayed in children with ADHD, but on the other, a higher sleep pressure likely plays a role in this disease. Although these two hypotheses might appear to be independent from one another or even in open contradiction, upon closer study, they may represent two faces of the same phenomena. Indeed, sleep disorders during brain development, carefully evaluated and described in our dataset, might have interfered with both sleep quality (causing an increased sleep pressure) and with brain maturation, especially when chronic and early-onset. If confirmed, these results could guide future clinical research in ADHD, favoring the investigation of sleep disorders and their early therapeutic treatment.

## Figures and Tables

**Table 1:** Clinical and instrumental sleep data in the attention-deficit/hyperactivity disorder (ADHD) group and in the healthy control group.

	ADHD (*n* = 30)			CONTROL (*n* = 23)				
	M ± SD	Median	Min–Max	M ± SD	Median	Min–Max		
Age (y)	10.48 ± 2.06	10.33	7.80–13.83	10.15 ± 1.56	10.00	7.92–13.67		
BMI (kg/m^2^)	18.71 ± 4.43	17.05	13.60–34.20	17.43 ± 2.71	17.10	13.80–23.60		
CPRS-R (tot)	76.11 ± 10.78	78	59–99					
WISC-IV (tot)	100.19 ± 9.02	98	84–114					
K-SADS-PL (tot)	2.93 ± 0.26	3	2–3					
Sex	Male		*n* = 21	Male		*n* = 12		
ADHD Subtypes	Inattentive		*n* = 6					
	Hyperactive		*n* = 2					
	Combined		*n* = 22					
Sleep Phenotypes	Epileptic EEG abnormalities		*n* = 10					
	Sleep onset insomnia		*n* = 5					
	PLMI > 5 events/hour		*n* = 8					
	OSAS		*n* = 15					
	Narcoleptic-like		*n* = 4					
**v-PSG ^§^**								
	**M ± SD**	**Median**	**Min–Max**	**M ± SD**	**Median**	**Min–Max**	**P**	**ES**
TIB (min)	493.40 ± 39.81	493.82	384.73–568.77	503.44 ± 41.04	506.55	390.00–567.80	0.377	
TST (min)	408.96 ± 58.73	423.00	215.95–478.50	430.69 ± 52.02	440.42	275.27–499.93	0.17	
SL (min)	29.72 ± 23.39	22.72	1.35–81.87	23.08 ± 14.32	23.98	3.29–58.58	0.578 *	
REML (min)	135.47 ± 56.75	116.38	55.50–299.57	111.01 ± 45.55	93.08	57.40–252.39	0.066 *	
WASO (min)	54.71 ± 55.31	28.58	7.24–216.45	50.84 ± 45.01	31.98	10.11–199.67	0.787	
SE (%)	83.12 ± 11.73	86.23	48.49–97.45	85.74 ± 9.44	89.02	55.22–97.45	0.388	
N1 (min)	23.16 ± 8.85	21.66	9.50–39.50	24.93 ± 10.48	23.70	11.00–56.01	0.512	
N1 (%TST)	5.89 ± 2.84	5.28	2.03–15.03	5.86 ± 2.62	5.49	2.89–12.36	0.971	
N2 (min)	151.47 ± 38.84	153.50	17.00–199.88	160.40 ± 24.71	165.48	93.00–199.70	0.343	
N2 (%TST)	36.51 ± 7.97	37.07	7.87–49.67	37.31 ± 4.51	35.46	29.58–46.87	0.668	
N3 (min)	141.45 ± 26.35	137.35	104.50–195.00	147.70 ± 19.14	144.50	109.50–181.96	0.345	
N3 (%TST)	35.12 ± 7.34	33.38	24.65–56.49	34.52 ± 4.08	34.35	27.20–42.47	0.727	
REM (min)	92.87 ± 23.56	97.25	44.50–129.13	97.66 ± 28.84	101.00	39.00–161.50	0.512	
REM (%TST)	22.48 ± 3.68	22.20	15.18–28.30	22.30 ± 4.82	22.01	14.03–33.00	0.881	
AI (n/h)	13.48 ± 3.44	13.90	7.42–19.67	12.74 ± 3.1	12.67	6.96–18.15	0.422	
AHI (n/h)	2.04 ± 1.70	1.40	0.00–6.90	0.67 ± 0.8	0.40	0.00–3.10	0.002	−1.002
PLMI (n/h)	2.96 ± 2.81	2.80	0.00–9.60	2.74 ± 3.55	1.50	0.00–12.70	0.807	

AI, arousal index; AHI, apnea-hypopnea index; BMI, body mass index; CPRS-R, Conners’ Parent Rating Scale—Revised; K-SADS-PL, Schedule for Affective Disorders and Schizophrenia for School-Age Children Present and Lifetime Version; N1, non-REM sleep stage 1; N2, non-REM sleep stage 2; N3, non-REM sleep stage 3; OSAS, Obstructive sleep apnea syndrome (based on AHI >1 event/hour and at least one among snoring, labored sleep breathing and sleepiness); PLMI, periodic limb movements index; REM, rapid eye movement; REML, REM latency; SE, sleep efficiency; SL, sleep latency; TIB, time in bed; TST, total sleep time; WASO: wakefulness after sleep onset; WISC-IV, Wechsler Intelligence Scale for Children—IV. ES: effect size; M: median, min: minutes n: number; n/h: number/hour; P: *p*-value resulting from to 2-tailed independent *t*-test statistics unless otherwise specified (* Mann-Whitney U Test); SD: standard deviation. ^§^ only 29 patients considered as for 1 child with ADHD only the first sleep cycle was available.

## Data Availability

The data presented in this study are available on request from the corresponding author.

## Supplementary Materials

The following are available online at https://www.mdpi.com/article/10.3390/children9020197/s1, Figure S1: Topographical distribution of all frequency bands based on whole-night non-rapid eye movement (NREM) sleep stage 2 (N2) and stage 3 (N3) in the attention-deficit/hyperactivity disorder (ADHD) group and in healthy control group, Figure S2: Topographical distribution of all frequency bands based on whole-night non-rapid eye movement (NREM) sleep stage 3 (N3) in the attention-deficit/hyperactivity disorder (ADHD) group with and without the obstructive sleep apnea/hypo-apnea phenotype, Figure S3: Spectral power density average over all artifact free epochs in the attention-deficit/hyperactivity disorder (ADHD) group and in the healthy control group during whole-night non-rapid eye movement (NREM) sleep stage 3 (N3), Figure S4: Topographical distribution of alternative frequency bands based on whole-night non-rapid eye movement (NREM) sleep stage 3 (N3) in the attention-deficit/hyperactivity disorder (ADHD) group and in the healthy control group, Figure S5: Topographical distribution of all frequency bands based on whole-night rapid eye movement (REM) sleep in the attention-deficit/hyperactivity disorder (ADHD) group and in healthy control group, Figure S6: Topographical distribution of all frequency bands based on wake before sleep onset in the attention-deficit/hyperactivity disorder (ADHD) group and in healthy control group.
