# Supplementary material for: Sleep Power Topography in Children with Attention Deficit Hyperactivity Disorder (ADHD)
Source: Children (Basel). 2022 Feb 3;9(2):197. doi: 10.3390/children9020197 (PMC8870029; doi:10.3390/children9020197)
Supplement: Supplementary file 1 [file children-09-00197-s001.zip › children-1548555-supplementary.pdf]

Supplementary Information

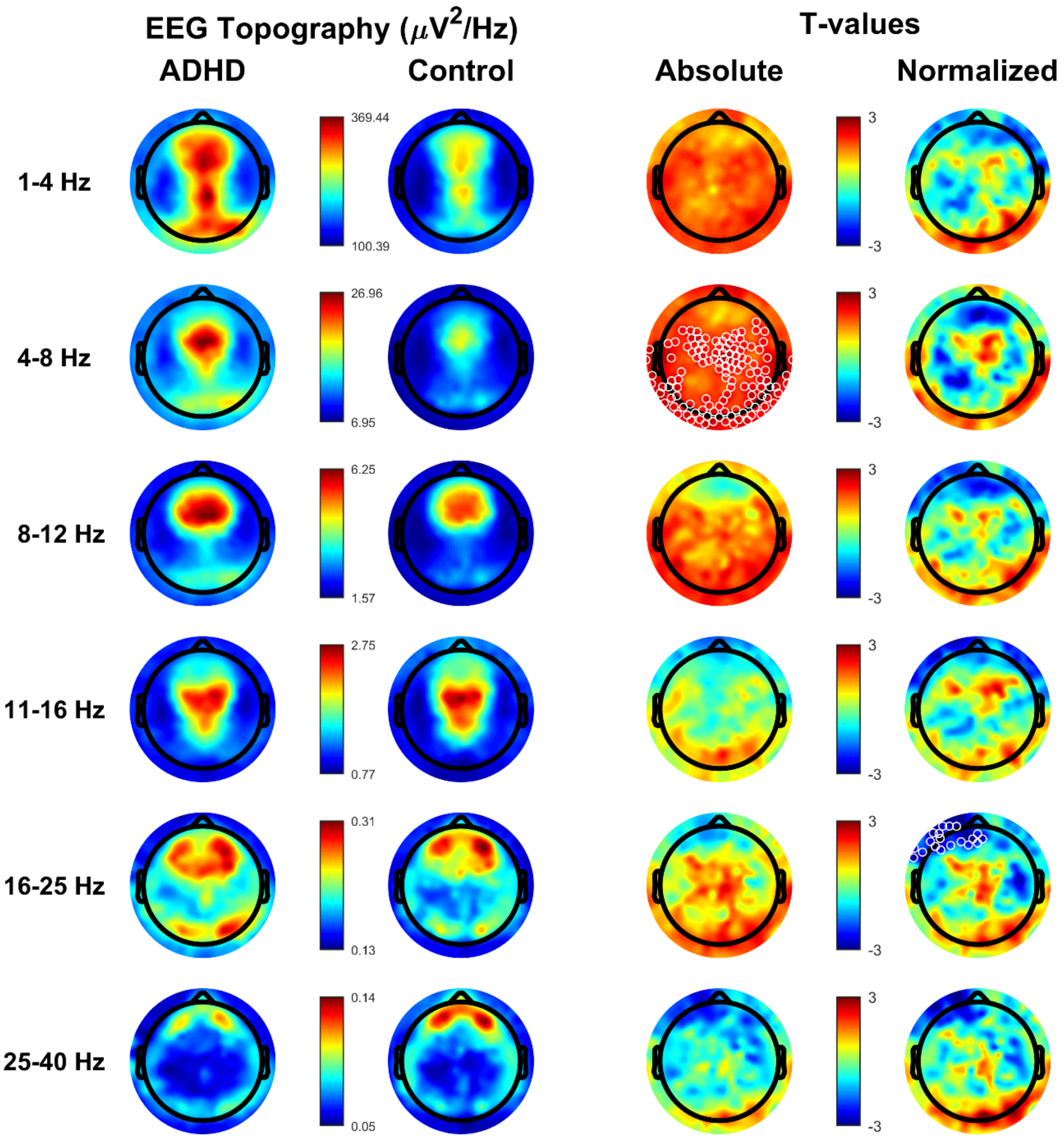

Supplementary Figure S1. Topographical distribution of all frequency bands based on whole-night non-rapid eye movement (NREM) sleep stage 2 (N2) and stage 3 (N3) in the attention-deficit/hyperactivity disorder (ADHD) group and in healthy control group.

Values are color coded and plotted on the planar projection of the hemispheric scalp model. Rows represent frequency bands of interest as indicated: Slow wave activity (SWA, 1-4 Hz), Theta (4-8 Hz), Alpha (8- 12 Hz), Sigma (11-16 Hz), Beta (18-25 Hz), Gamma (25-40 Hz). First and second column: average NREM sleep electroencephalographic (EEG) topographies across frequency bands for children with ADHD and healthy control matches, respectively. Maxima are shown in red, minima in blue. Third and fourth column: single electrode t-value (two-tailed, unpaired) maps for the comparison between ADHD and control subjects in terms of absolute and normalized (using the z-score across all electrodes) power, respectively. A decrease in EEG power in patients with ADHD relative to healthy controls (ADHDs < controls) is represented in blue, an increase (ADHD > controls) in red. White circles indicate significant channels ($p < 0.05$*, cluster-size correction).*

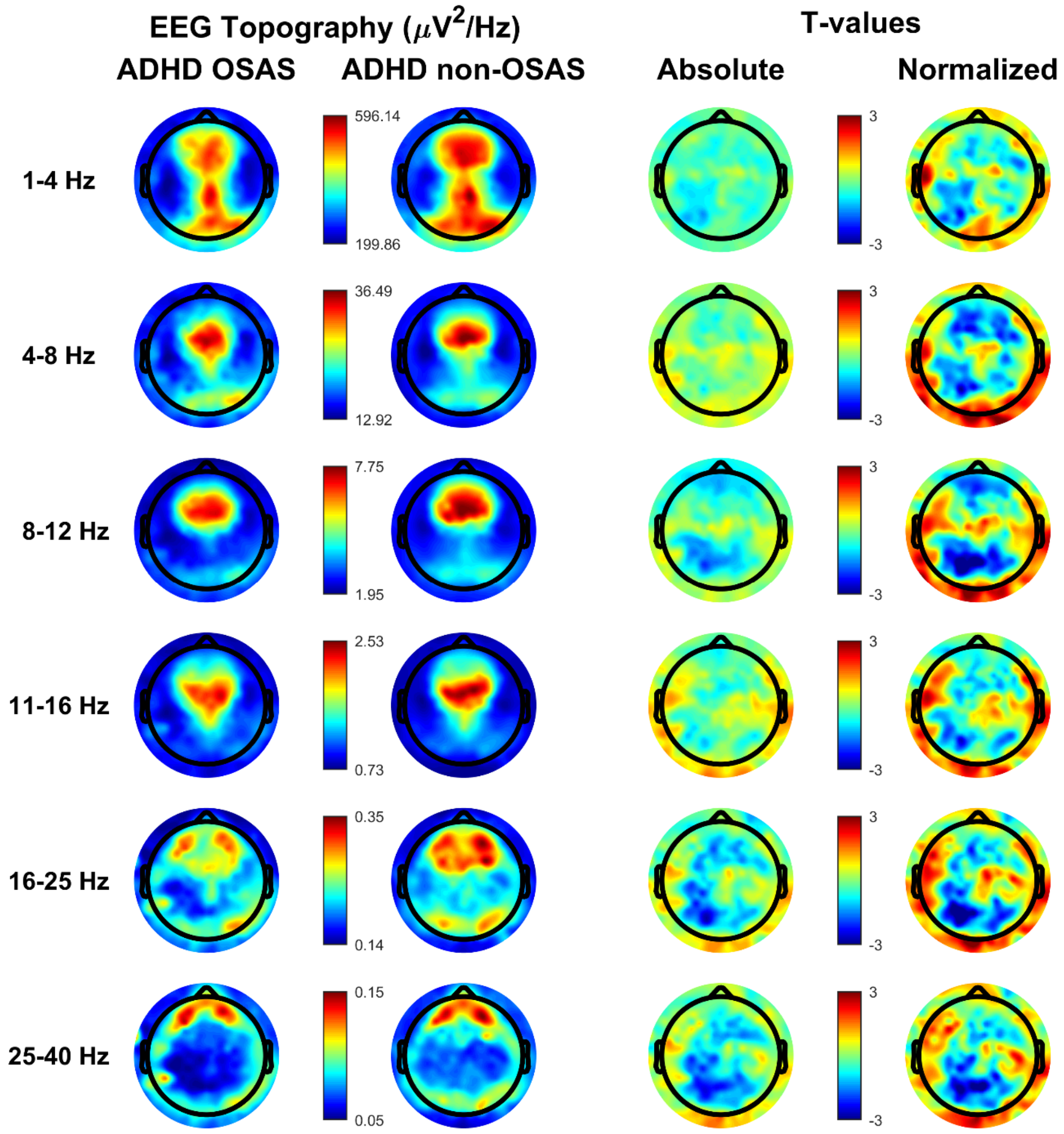

Supplementary Figure S2. Topographical distribution of all frequency bands based on whole-night non-rapid eye movement (NREM) sleep stage 3 (N3) in the attention-deficit/hyperactivity disorder (ADHD) group with and without the obstructive sleep apnea/hypo-apnea phenotype.

Values are color coded and plotted on the planar projection of the hemispheric scalp model. Rows represent frequency bands of interest as indicated: Slow wave activity (SWA, 1-4 Hz), Theta (4-8 Hz), Alpha (8- 12 Hz), Sigma (11-16 Hz), Beta (18-25 Hz), Gamma (25-40 Hz). First and second column: average N3 sleep electroencephalographic (EEG) topographies across frequency bands for children with ADHD with and without an obstructive sleep apnea/hypo-

apnea phenotype, respectively. Maxima are shown in red, minima in blue. Third and fourth column: single electrode t-value (two-tailed, unpaired) maps for the comparison between ADHD and control subjects in terms of absolute and normalized (using the z-score across all electrodes) power, respectively. A decrease in EEG power in patients with ADHD with the apnea/hypo-apnea phenotype relative to in patients with ADHD without the apnea/hypo-apnea phenotype is represented in blue, an increase in red. White circles indicate significant channels ($p < 0.05$, cluster-size correction).

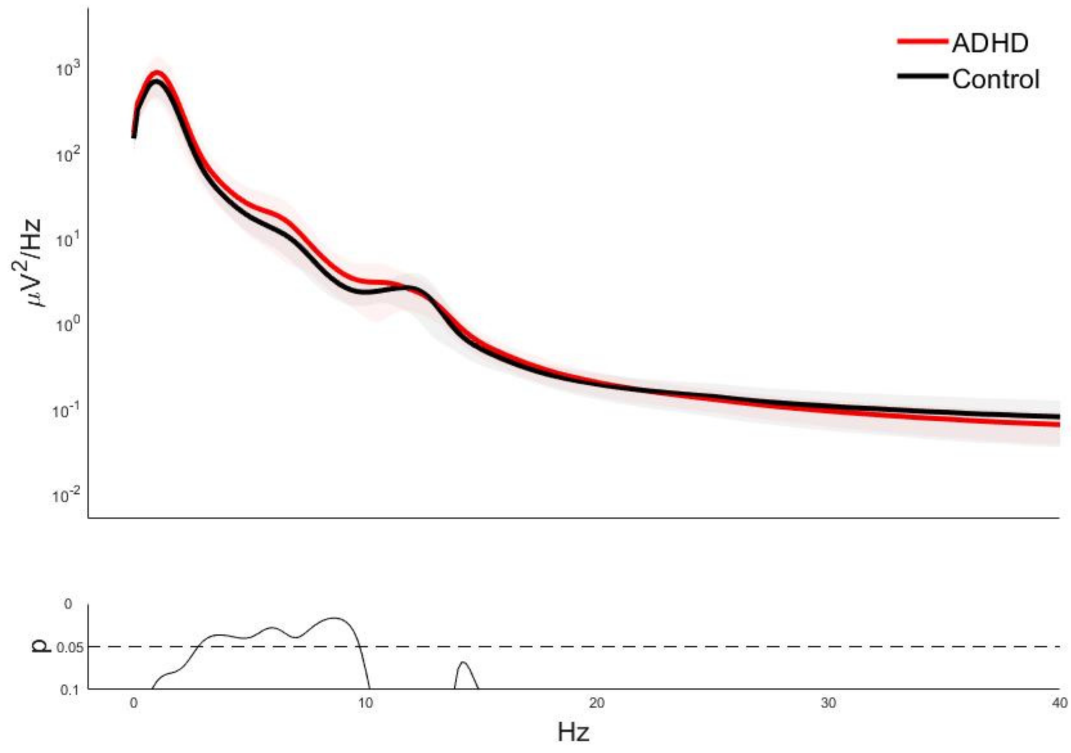

Supplementary Figure S3. Spectral power density average over all artifact free epochs in the attention-deficit/hyperactivity disorder (ADHD) group and in the healthy control group during whole-night non-rapid eye movement (NREM) sleep stage 3 (N3).

Shaded area represents standard error of the mean (SEM). Bottom graph shows p-values, reaching statistical significance between 3 and 10 Hz.

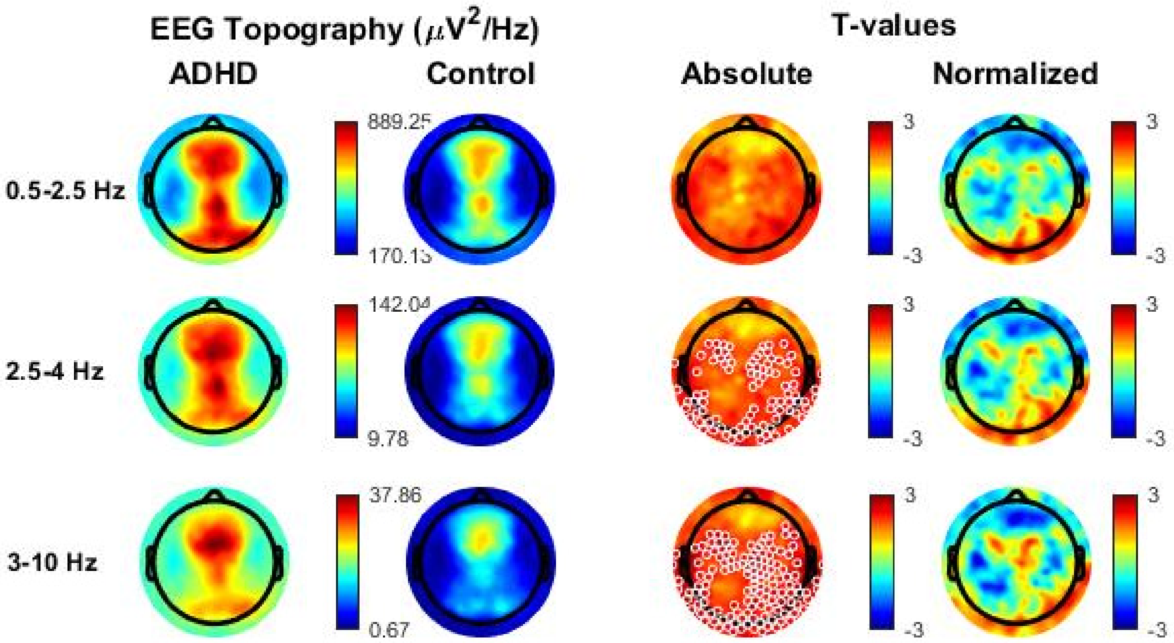

Supplementary Figure S4. Topographical distribution of alternative frequency bands based on whole-night non-rapid eye movement (NREM) sleep stage 3 (N3) in the attention-deficit/hyperactivity disorder (ADHD) group and in the healthy control group.

Values are color coded and plotted on the planar projection of the hemispheric scalp model. Rows represent frequency bands of interest as indicated on the left. The first two rows focused respectively on the low-delta (0.5-2.5 Hz) and high-delta (2.5-4 Hz) frequency ranges. The 3-10 Hz band was selected from the inspection of the average power spectra. First and second column: average N3 sleep EEG topographies across frequency bands for children with ADHD and healthy control matches, respectively. Maxima are shown in red, minima in blue. Third and fourth column: single electrode t-value (two-tailed, unpaired) maps for the comparison between ADHD and control subjects in terms of absolute and normalized (using the z-score across all electrodes) power, respectively. A lower EEG power in patients with ADHD relative to healthy controls (ADHDs < controls) is represented in blue, a higher power (ADHD > controls) in red. White circles indicate significant electrodes ($p < 0.05$, cluster-size correction).

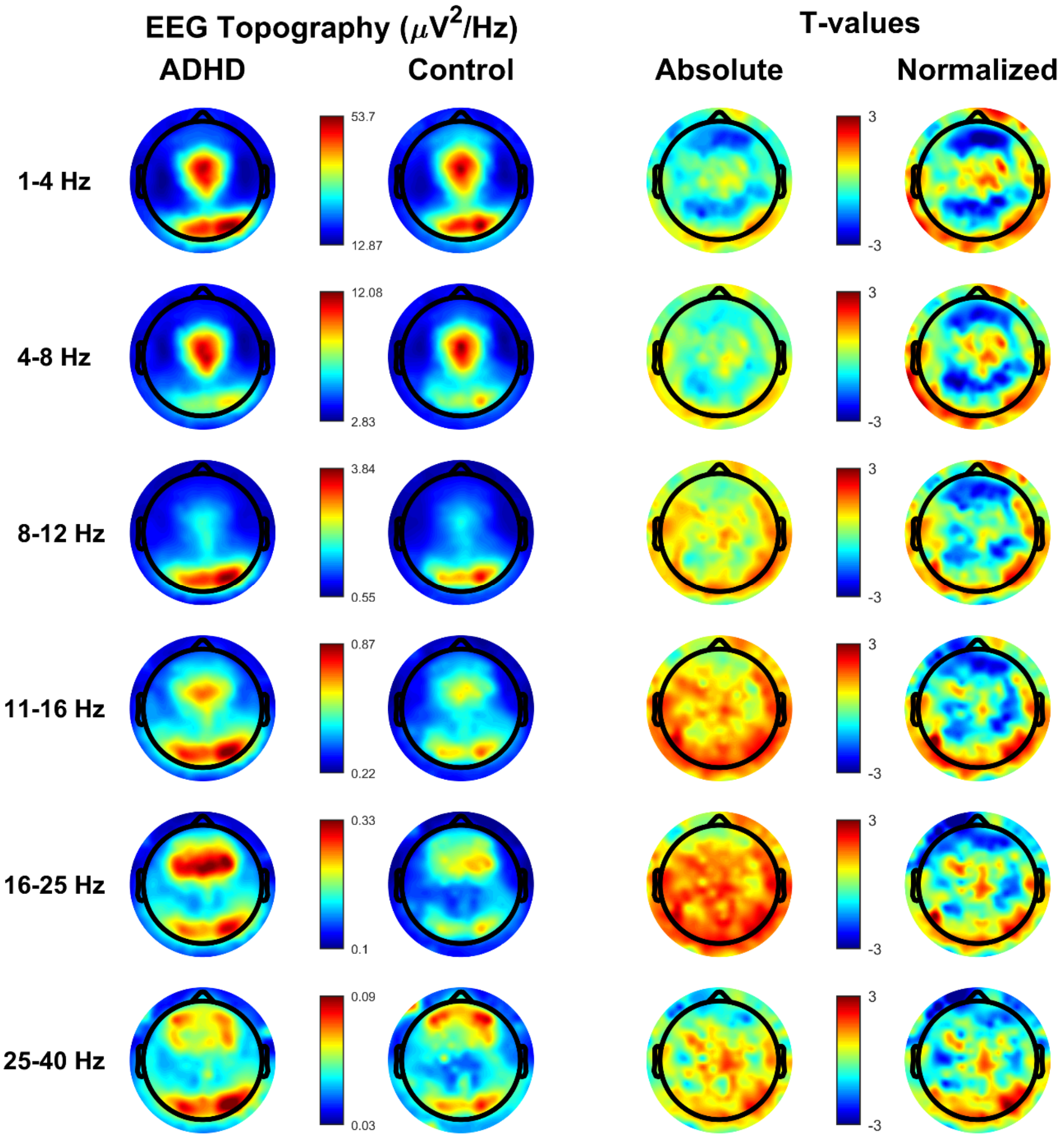

Supplementary Figure S5. Topographical distribution of all frequency bands based on whole-night rapid eye movement (REM) sleep in the attention-deficit/hyperactivity disorder (ADHD) group and in healthy control group.

Values are color coded and plotted on the planar projection of the hemispheric scalp model. Rows represent frequency bands of interest as indicated: Slow wave activity (SWA, 1-4 Hz), Theta (4-8 Hz), Alpha (8- 12 Hz), Sigma (11-16 Hz), Beta (18-25 Hz), Gamma (25-40 Hz). First and second column: average REM sleep electroencephalographic (EEG) topographies across frequency bands for children with ADHD and healthy control matches, respectively. Maxima are shown in red, minima in blue. Third and fourth column: single electrode t-value (two-tailed, unpaired) maps for the

comparison between ADHD and control subjects in terms of absolute and normalized (using the z-score across all electrodes) power, respectively. A lower EEG power in patients with ADHD relative to healthy controls (ADHDs < controls) is represented in blue, a higher power (ADHD > controls) in red. White circles indicate significant electrodes ($p < 0.05$ cluster-size correction).

Given that the between-subject variability in the number of REM cycles and the fact that the first REM cycle was often very short, we repeated the same analysis on the second/third cycles in 27 patients and 23 control subjects, and again found no significant differences between groups.

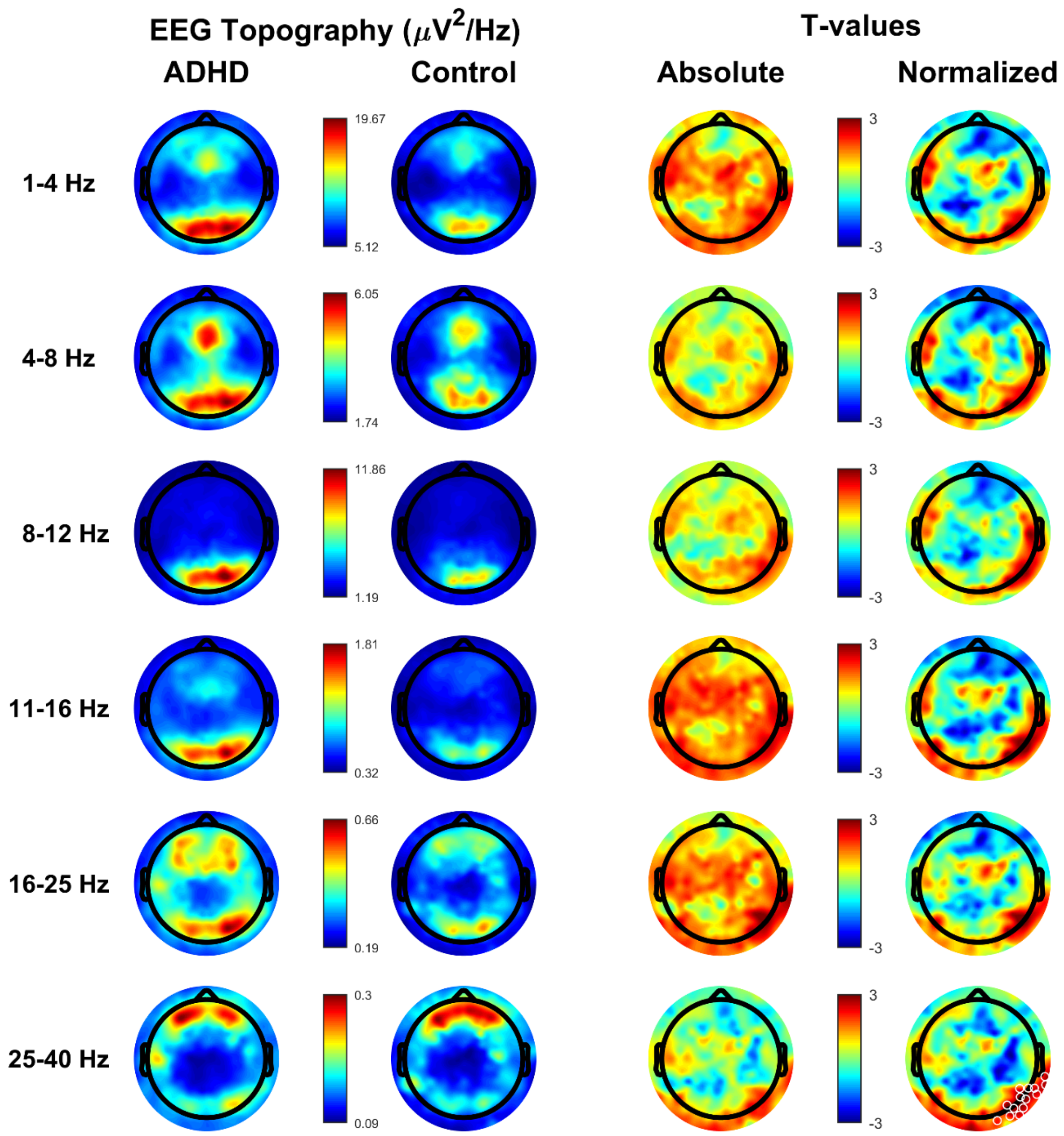

Supplementary Figure S6. Topographical distribution of all frequency bands based on wake before sleep onset in the attention-deficit/hyperactivity disorder (ADHD) group and in healthy control group.

A comparison of pre-sleep signal power between ADHD and control children was performed using all subjects who had a sufficient amount of artifact-free data in the interval comprised between 50 and 10 min before sleep onset (ADHD N = 26, HD N = 18). Three patients and 4 control subjects did not have artifact-free segments of wakefulness before sleep onset longer than 5 minutes and were therefore excluded.

Values are color coded and plotted on the planar projection of the hemispheric scalp model. Rows represent frequency bands of interest as indicated: Slow wave activity (SWA, 1-4 Hz), Theta (4-8 Hz), Alpha (8- 12 Hz), Sigma (11-16 Hz), Beta (18-25 Hz), Gamma (25-40 Hz). First and second column: average wake before sleep onset electroencephalographic (EEG) topographies across frequency bands for children with ADHD and healthy control matches, respectively. Maxima are shown in red, minima in blue. Third and fourth column: single electrode t-value (two-tailed, unpaired) maps for the comparison between ADHD and control subjects in terms of absolute and normalized (using the z-score across all electrodes) power, respectively. A lower EEG power in patients with ADHD relative to healthy controls (ADHDs < controls) is represented in blue, a higher EEG power (ADHD > controls) in red. Significant electrodes indicate as white dots (p ≤ 0.05 using statistical nonparametric mapping suprathreshold cluster testing).
